# Autogenous dentin for socket preservation: a narrative review of clinical outcomes compared to spontaneous healing

**DOI:** 10.3389/fdmed.2025.1714903

**Published:** 2025-11-14

**Authors:** Petr Jalůvka, Jan Šrubař, Martin Starosta

**Affiliations:** 1 Department of Dentistry, Faculty of Medicine, University of Ostrava, Ostrava, Czechia; 2 Department of Oral and Maxillofacial Surgery, University Hospital Ostrava, Ostrava, Czechia; 3 Department of Dentistry, University Hospital Ostrava, Ostrava, Czechia

**Keywords:** autologous dentin, autogenous dentin, socket preservation, ridge augmentation, spontaneous healing, tooth extraction

## Abstract

Socket preservation after tooth extraction is a critical procedure to maintain alveolar ridge dimensions for future prosthetic rehabilitation. Autogenous dentin has emerged as a promising graft material due to its biocompatibility, osteoconductivity, and similarity in composition to bone. A systematic search of MEDLINE (PubMed), Scopus, and Web of Science was conducted in January 2025 using a defined MeSH-based strategy. The search was limited to human clinical studies published in the past ten years. Only studies directly comparing groups with autogenous dentin grafts and spontaneous healing without augmentation were included. Six studies met the criteria and were included in the final review, all confirming the safety and biocompatibility of autogenous dentin. Histological evaluations showed active bone formation around dentin particles and high osteoblastic activity without inflammatory response. Cone beam Computed Tomography (CBCT) analysis, performed in most studies, revealed significantly better preservation of ridge dimensions in autogenous dentin matrix (ADM)-augmented sites, especially in the coronal third of the socket. One study highlighted successful outcomes even in periodontally compromised molar extractions. Despite the general agreement among studies, further research (ideally with standardized protocols) is needed to confirm long-term efficacy in diverse clinical scenarios.

## 1 Introduction

Tooth loss is a common consequence of caries, trauma, or advanced periodontal disease. After tooth extraction, the alveolar bone undergoes partial resorption over time, which leads to a substantial reduction in its volume (1). This is particularly obvious in the dimensional changes in the buccal-lingual width, which decrease by up to 50%. This bone loss is most pronounced in the first 3 months post-extraction; the resorption process, however, continues even after that period, with an average annual loss of 0.5%–1.0% (2). Bone resorption is particularly significant in the frontal and premolar areas of the jaw, in the region of the thin buccal bone cortex (3). Various surgical procedures aiming to maximize the preservation of bone volume were described and validated in the literature (4). The use of methods supporting socket preservation (also called alveolar ridge preservation or alveolar ridge augmentation) facilitates subsequent implant placement or other esthetic restoration techniques (5).

**Figure 1:**
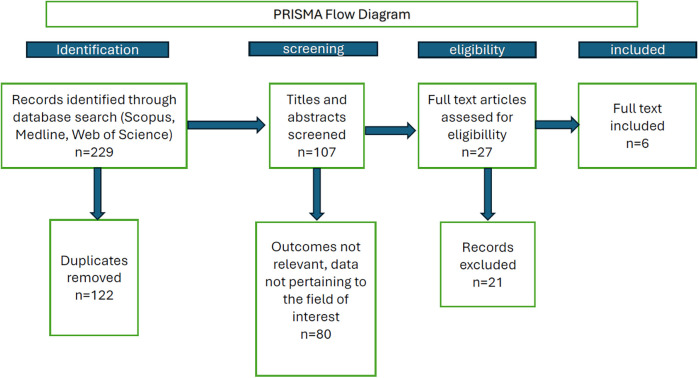
PRISMA diagram of the search strategy and selection of publications.

Socket preservation is a technique-dependent process, the outcome of which depends not only on the approach used but also on the skill of the dentist/oral surgeon. The extraction technique is suspected to be the key factor affecting the degree of bone resorption after surgery (6). Besides, the blood clot stability and sealing of the extraction site have been reported to positively affect the healing process (7). The biomaterials used in socket preservation possess osteoconductive properties, promoting bone growth while preserving space for it. The materials, however, differ in their performance from the perspectives of graft resorption and new bone formation.

A wide variety of materials have been shown to yield better results than natural healing (8). Graft materials can be classified into four categories: autograft (bone from the same patient), allograft (bone from another human), xenograft (bone from another species), and alloplast (synthetic material) (9). Allografts are, however, costly, and xenografts were shown to lack osteoinductive properties. Creating an autogenous dentin matrix from the patient's own extracted tooth is another interesting approach to acquiring a viable material for the augmentation procedure. Many researchers have explored the possibility of using (chairside-prepared) partially demineralized autogenous dentin matrix in regenerative procedures, and the effectiveness of this approach has been demonstrated previously (9, 10). A major advantage lies in the fact that the composition of the tooth-derived demineralized dentin matrix is similar to that of the bone. Moreover, the method appears to be associated with a minimal risk of disease transmission and is relatively cost-effective (10).

As mentioned above, the structure and composition of dentin closely resemble that of bone, consisting of organic components (20%), hydroxyapatite (70%), and body fluid (10%). Dentin is believed to exhibit high osteoconductivity since it is a natural mineralized hydroxyapatite-containing tissue. Moreover, the dentin matrix is expected to exhibit osteoinductive properties thanks to the presence of bone morphogenetic proteins (BMPs) (11). Although the composition of BMPs derived from human dentin differs from that derived from BMPs, the function of both types in the body is similar (12). Some studies using demineralized dentin matrix have, however, shown that although the material possesses excellent biocompatibility, its effectiveness in supporting bone formation is lower than that of bone-derived materials. On the other hand, animal studies have shown demineralized dentin matrix (DDM) to be not only biocompatible, but also osteoinductive, similar to demineralized bone matrix (11). Dentin was also shown to contain several growth factors, including transforming growth factor beta (TGF-*β*), insulin-like growth factor-II (IGF-II), and bone morphogenetic protein-2 (BMP-2), which could be of pivotal importance during healing, can be utilized in socket preservation (13).

The particle size is another factor that needs to be taken into account when using DDM to support bone formation/regeneration. Smaller particles have been associated with enhanced cellular responses and increased osteoinductive properties, while larger particles can act as a scaffold, facilitating the ingrowth of new bone and promoting its formation within the defect site (14).

As mentioned above, most studies using dentin for socket preservation perform (at least partial) demineralization prior to its use. Full demineralization is, however, associated with an extended preparation time and reduces the available graft volume. Besides, prolonged acid exposure can result in the depletion of growth factors and the collapse of the 3D architecture of dentin. Moreover, results obtained using fully or partially demineralized tooth bone grafts are very similar, which speaks in favor of the simpler method, i.e., partial demineralization (13).

All of the above indicate that the use of autogenous dentin in socket preservation is a promising approach that can greatly benefit patients after tooth extraction, ensuring sufficient bone volume for subsequent implant placement. In this narrative review, we aimed to provide a clear overview of studies comparing the use of autogenous dentin graft for socket preservation after tooth extraction with a control group consisting of patients in whom the alveolar ridge was left to heal spontaneously.

## 2 Materials and methods

This study followed a structured MeSH-based search protocol. A comprehensive search was performed in the MEDLINE (PubMed), Scopus, and Web of Science (Clarivate) databases using the following search string: (autologous OR autogenous) AND dentin AND [(socket OR ridge) AND (augmentation) OR (preservation)]. The publication date range was limited to the past 10 years to ensure obtaining the maximum amount of relevant sources (as the use of autogenous dentin is a relatively new method, extending the search further into the past would likely only yield irrelevant results). The search was completed by January 13, 2025.

The database searches yielded 81 (MEDLINE), 91 (Scopus), and 57 (Web of Science) records, respectively (229 records in all), 123 of which were duplicates. After removing these duplicates, 107 records were retained. Subsequently, titles and abstracts were scanned, and only original research papers in English with human subjects were selected for full reading. Such papers were read by two researchers; where conflict on whether or not the particular paper is suitable for inclusion arose, the intention was to consult the senior (corresponding) author to decide; nevertheless, no such instance occurred. Risk of bias was assessed using the Cochrane ROB 2 tool.

As this review aimed to evaluate the alveolar bone healing for prosthetic restoration, and third molars are typically not candidates for such restoration, studies on third molars were excluded from the search results. In addition, only clinical studies comparing a control group (spontaneous healing without augmentation) with a treatment group (autogenous dentin grafts) were included in the study. Only six publications met those final criteria and were selected for the review (15–20), see the PRISMA diagram in Figure 1.

## 3 Results

Details of patient populations in the 6 studies included in our review are presented in Table 1, the applied methods are presented in Table 2. The risk of bias evaluation, presented in Table 3, showed that even though most of the studies had no critical concerns, none of them could be evaluated as “low risk of bias” overall.

**Table 1:** Population characteristics and inclusion/exclusion criteria employed in the six studies selected for the review.

Reference	Number of patients (Men/Women)	Excluded after surgery	Number of teeth (sockets)	Number of teeth (sockets) after exclusion	Age	Inclusion criteria	Exclusion criteria
Yüceer-Cetiner et al.	9 (4/5)	0	57	57	31–62 years	18+ with no systemic health condition (ASA I, II), adequate restorative space for implant placement	Teeth with deep decay of root canal filling, acute infection, pregnancy or lactation, low compliance
Yang et al.	32 (15/17)	13 excluded before surgery	32	32	21–72 years	18+ with no systemic health condition, presence of periodontally hopeless molar with severe bone loss that required extraction, full mouth plaque index lower than 20% and full mouth bleeding index lower than 25%.	Active periodontitis, pregnancy or lactation, alcoholism, radiotherapy in the head and neck area, medications or treatments with an effect on healing in general, metabolic disorders or osteoporosis, smoking >10 cigarettes a day
Hussain et al.	45	13	32	29 (11W/18M)	Mean 35 ± 11.31 years in control group/36, 29 ± 11.72 in study group	18+ needing extraction of maxillary non molar tooth	Periodontitis, decompensated systemic disease, pregnancy, lactation, osteoporosis, smoking or alcohol abuse, history of radiotherapy or immunosuppressants, use of bisphosphonates of systemic corticosteroids, absence of buccal or palatal bone plate
López Sacristán et al.	30 (N/A)	8	60	44	21–62 years	18+, partial or complete tooth loss, eligibility for dental implant placement	History of uncontrolled systemic disease, clotting disorder or anticoagulant use, bisphosphonate treatment, severe immune system compromise, mental instability, alcohol or drug abuse
del Canto-Diaz et al.	9 (4/5)	3	18	12	39–62 years	18+ needing extraction of two single-rooted teeth for periodontal reasons, root caries or fractures and consent with implant placement	Endocrine/metabolic disorder possibly affecting bone regeneration, presence of acute or chronic process (general or local), bisphosphonates therapy, smokers 10+ cigarettes, at least one missing alveolar wall after extraction
Isola et al.	14 (6/8)	0	28 non molar maxillary	28	37–62 years	18+, no systemic disease, absence of active periodontal disease, scheduled for implant placement	Pregnancy, previous or current radiotherapy or immunosupressant therapy, medication with antiinflammatory or immunosuppressive drugs, smoking, history of alcohol abuse, absence of occluding dentition in the socket area, more than 50% loss of buccal bone plate after extraction

**Table 2:** Techniques and methods employed in the six studies selected for the review.

Reference	Primary technique of ADM	Control Group Technique	Evaluation	Implant placement	Antibiotics	Preparation of ADM	Suture material	Other material used
Yüceer-Cetiner et al.	undermineralized autogenous dentin graft (Group D—20 sockets)	spontaneous healing (Group C -16 sockets)	Histology	57 implants, one year follow up, 100% success rate	Yes	Smart dentin grinder, KometaBio, Israel, 300–1,200*μ*m particles	Silk 3/0	Resorbable membrane (Geistlich Bioguide) in groups D and DP
Yang et al.	Autogenous partially demineralized dentin matrix (APDDM)	Spontaneous healing (16 sockets)	Histology + CBCT	32 implants were placed, one case in study group needed extra augmentation technique during implant placement, 5 cases in the control group	Yes	Bonmaker (Korea dental solution), 425–1,200 μm particles	4/0 non-resorbable monofilament polyprophylene in test group	collagen sponge (Wuxi BIOT) in the study group
Hussain et al.	Autologous dentin material (14 sockets)	Spontaneous healing + suture (15 sockets)	Histology + CBCT	29 implants	Yes	Smart dentin grinder, Kometa Bio 300–1,200μm particles	not reported	gel foam Roeko Gelatamp in the study group
López Sacristán et al.	Autologous dentin material	Blood clot stabilization covered by collagen material and suture	Histology + CBCT + Densitometry	not reported	N/A	Smart dentin grinder KometaBio	5/0 monofilament	collagen membrane (Lyoplant, B.braun) for both groups
del Canto-Diaz et al.	freshly processed autologous dentin graft (6 sockets after exclusion)	Collagen membrane and suture (6 sockets after exclusion)	CBCT + Densitometry	One of the inclusion criteria but actual number of implants not mentioned	N/A	Smart dentin grinder, 300–1,200μm particles	5/0 monofilament	15 × 20 mm collagen membrane over the extraction site in both groups
Isola et al.	Tooth-derived mineralized dentin matrix graft (DDM)	Spontaneous healing covered by free gingival graft (FGG) and suture	Histology + intraoral radiographs + bucopalatal alveolar width measurement	28 implants	Yes	Tooth transformer Biomax	5/0 resorbable Vicryl plus	Free gingival graft taken from hard palate for both groups

**Table 3:** Results of bias evaluation in individual domains according to the ROB 2 tool.

Reference	D1	D2	D3	D4	D5	Overall judgement
Canto-Díaz et al. (2019)								
Hussain et al. (2023)								*Low risk*
Isola et al. (2022)								*Some concerns*
Yang et al. (2023)								*High risk*
López-Sacristán et al. (2024)								
Yüceer-Çetiner et al. (2021)								

D1, bias arising from the randomization process; D2, bias due to deviations from intended interventions; D3, bias due to missing outcome data; D4, bias in measurement of the outcome; D5, bias in selection of the reported result.

The earliest of the six studies (15) was a pilot study involving nine patients with bilateral extraction of non-molar single-rooted teeth, comparing dimensional changes in the alveolar bone between the sides, with ADM applied to one extraction site only. Both extraction sites were covered by a collagen membrane and sutured. At 16 weeks after the surgery, the dimensional contraction of the ADM-augmented sockets was significantly lower than in the control teeth, both in vertical and horizontal dimensions. Densitometric analysis showed no significant differences between the control and study groups, with the exception of the coronal area of the socket, where bone density values were higher in the augmented sockets. However, one-third of the patients entering the study did not attend the control examination and could not be evaluated. It is worth noting that significant differences were found despite the very small study group in this pilot study. The second study (20) (in chronological order) included two experimental groups and one control group. Both experimental groups used autologous dentin; in one of them, autologous dentin was combined with platelet-rich fibrin (PRF). The outcomes were evaluated using histopathology, immunohistochemistry, and electron microscopy methods. The results showed significant differences between the control and experimental groups; moreover, some values differed between the experimental groups as well. Higher vascularisation and a higher percentage of new bone formation were observed in the group with PRF; on the other hand, a higher representation of new connective tissue was observed in the group with non-augmented ADM. Although the number of patients was relatively small (nine patients), a total of 57 extraction sites were evaluated. The material for histological examination was harvested during the preparation for implant placement.

The study by Isola et al. (18) stood out by using free gingival graft (FGG) to cover the extraction site in both groups (control group and experimental group in which autologous dentin was used; 14 patients, 28 teeth). Only extraction sites after maxillary non-molar teeth were considered in their study. The outcomes were evaluated by histology using samples harvested during implant placement, as well as by manual measurements of bone dimensions before extraction and 16 weeks post-extraction. While the authors found no significant dimensional differences in the ridge between the groups (which might have been at least partially due to the use of a manual measurement technique), histological analysis revealed a significantly greater amount of newly formed vital bone in the ADM group.

Yang et al. (17) were the only ones to use autogenous dentin from teeth with severe periodontal destruction (although in some cases, they supported it with dentin from another extracted tooth from the same patient). Their study focused exclusively on molar extraction sites. The experimental group received ADM grafts, while the control group was left to heal spontaneously, without any suturing or material placement. The evaluation was based on histological examination of samples harvested during implant placement and on the comparison of CBCT imagery immediately before extraction and 4 months after it (before implant placement). The results demonstrated a statistically significant difference in volumetric changes at the extraction site between groups, with the ADM group showing a greater volume of newly formed bone compared with the controls.

Hussain et al. (16) evaluated the effectiveness of autogenous dentin in alveolar ridge preservation following the extraction of maxillary non-molar teeth with intact alveolar crest bone. In both the control and treatment groups, the soft tissues were sutured after extraction. ADM in the test group was covered with a collagen cap and sutured. The authors evaluated CBCT results, measured the gingival thickness using an acrylic template made before extraction, and performed a histological examination of samples collected during implant placement. Only 29 out of 45 patients completed this study. Still, the analysis revealed a statistically significant difference in the magnitude of dimensional changes of the extraction site, but no significant difference in the total bone volume in histological samples was detected.

The last of the included studies (19) was a split-mouth study, i.e., all patients underwent autotransplantation of at least two single-rooted teeth, with one tooth being augmented using ADM and the other only with clot stabilization. Both groups received a collagen cap and soft tissue suturing. The outcomes were analyzed using CBCT performed at three time points (immediately after surgery, after 8 and 16 weeks) and by histological examination from samples taken during implant placement (only ADM-augmented teeth). The results demonstrated highly significant differences in the coronal third of the alveolar bone, while no differences were detected in the apical third of the socket. The authors considered autologous dentin to be an ideal slow-resorbing material for socket preservation.

The summary results of CBCT evaluation on the baseline (i.e., immediately before or immediately after the extraction) and during the follow-up (typically 16 weeks later) are summarized in Table 4.

**Table 4:** Changes in the alveolar bone width according to CBCT measurement at the baseline (immediately pre- or post-operatively) and during follow-up examination.

Reference	Parameter	Pre-Op or immediately Post-Op*		p16 weeks Post-Op		*p*
		Dentin	Control	Dentin	Control	
del Canto-Diaz et al.	VL-BBC 1 mm	3.14 ± 0.61	3.23 ± 0.60	2.68 ± 0.48	1.31 ± 1.63	*p* = 0.098
	VL-BBC 5 mm	3.22 ± 0.63	3.23 ± 0.86	3.23 ± 0.65	2.34 ± 1.42	*p* < 0.001
Hussain et al.	HW 2 mm**	7.93 ± 0.99	7.94 ± 1.17	6.46 ± 1.21	4.39 ± 0.70	*p* = 0.002
	HW 5 mm	9.06 ± 0.97	8.68 ± 1.10	7.82 ± 0.87	6.48 ± 0.91	*p* = 0.002
Yang et al.	HW 1 mm***	5.75 ± 5.97	7.98 ± 5.60	+4.50 ± 4.41^†^	−2.19 ± 3.21^†^	*p* < 0.001
	HW 5 mm***	13.52 ± 3.90	14.59 ± 3.34	+0.64 ± 3.14^†^	−1.18 ± 2.32^†^	*p* = 0.073
López Sacristán et al.	VL-BBC 1 mm	4.17 ± 0.65	4.22 ± 0.74	3.51 ± 0.63	2.90 ± 0.81	*p* < 0.001
	VL-BBC 5 mm	3.72 ± 0.58	3.90 ± 0.92	3.44 ± 0.67	3.22 ± 1.06	*p* > 0.050

VL-BBC = distance between vertical line (tooth axis) and buccal bone crest; HW = horizontal ridge width × mm below the most coronal aspect of the bone crest.

* Yang et al. only performed CBCT pre-operatively.

** Hussain et.al do not offer measurements at 1 mm below the alveolar crest.

*** The greater initial values are caused by the fact that only molars were used in that study.

† At the 4-month follow-up, only changes in bone width (mean ± SD) compared to the baseline are reported.

## 4 Discussion

Although all the included studies used autologous dentin as grafting material, none of the evaluation methods was applied across all of them. Histological examination was the most common method, with just the oldest pilot study (15) not using this evaluation. In all the remaining studies, samples of the bone tissue from the extraction site were taken during implant placement at 16 weeks [or, in one case, at three months (20)] post-extraction. In nearly all the studies, histology showed the formation of new bone surrounding the residual dentin particles. High osteoblastic activity was observed on the margins of the newly formed bone as well as on the surfaces of the dentin particles. No signs of inflammation or other foreign body processes were observed in any of the studies.

Prophylactic antibiotics (ATBs) were used in most studies (see Table 2); however, the only study not employing antimicrobial prophylaxis (19) yielded results practically identical to those using antibiotics. From this perspective, the impact of the use of antibiotics on the healing process in patients treated with ADM remains unclear. Still, this question warrants attention—while ATBs are expected to improve the procedural safety in everyday dental practice, their overprescription contributes to the growing problem of ATB resistance. Further research should, therefore, address this question.

**Figure 2:**
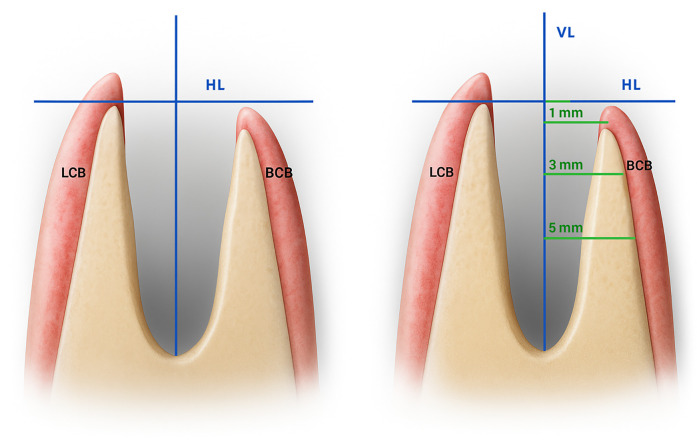
Measurement principle for socket preservation at multiple depths within the extraction site [adapted from (15)]: the distance between the lingual and buccal cortical bone is measured at 1 mm, 3 mm, and 5 mm apical depths relative to a horizontal reference line (HL) drawn on CBCT images. This line is defined by the most coronal point of the crestal bone and is oriented perpendicular to the vertical axis of the tooth. VL, vertical line (dental axis); HL, horizontal line; LCB, Lingual cortical bone; BCB, buccal cortical bone.

Radiological evaluation of the socket site using CBCT was the second most common method of bone mass evaluation. Of the four studies using CBCT, three studies (15, 17, 19) used a similar method of CBCT evaluation (with Del Canto-Díaz et al. (15) being the original paper introducing this methodology, see Figure 2). Typically, CBCT images taken before (16, 17) or immediately after (15, 19) the surgery were compared with those taken eight (15, 19) and/or 16 weeks post-surgery (15–17, 19). Although various techniques can be used to measure the alveolar ridge dimensions, CBCT is widely regarded as the gold standard for this purpose due to its high sensitivity and specificity (21). The comparison of results from CBCT analysis showed that the highest (statistically significant) differences between the groups with and without augmentation using autologous dentin were observed in the upper (coronal) third of the socket's vertical dimension.

The ideal timing of taking the CBCT is also debatable. Before extraction, CBCT is often performed to confirm the indication for extraction and aids in choosing the optimal (least traumatic) approach to extraction. On the other hand, post-extraction CBCT allows a more accurate evaluation of the socket's post-extraction condition as the bone wall (especially the buccal bone) can be damaged during the extraction. This consideration led several studies (15, 16, 18) to exclude cases with compromised socket walls, which would, obviously, not be possible based on the pre-extraction CBCT. From this perspective, it would be ideal to take CBCT imagery both before and after extraction; this would, however, raise concerns from the perspectives of the increased radiation burden to the patient and increased costs.

Del Canto-Díaz found a major reduction in the alveolar bone loss following the tooth extraction—the vertical loss was reduced from 16.9% of the initial alveolar height to only 4.2% in the ADM-augmented group (15). Similarly, López Sacristian et al. (19) detected significant differences favoring the ADM group in practically all measured parameters. Yang et al. (17), who applied the ADM augmentation in periodontally compromised teeth, found the VL-BCB width (see Figure 2) at 1 mm below the horizontal line to be increased by approx. 5 mm in the group using ADM 16 weeks after the extraction, while a decrease in this parameter by approx. 2 mm was observed in the control group. All studies consistently indicated that the most substantial differences between the control and ADM groups were found in the coronal third of the socket, with the impact of ADM diminishing apically. The last study employing CBCT analysis (16) used a similar methodology, with reference lines placed at 2 mm and 5 mm below the horizontal line. Moreover, they employed an acrylic plate to facilitate the evaluation of both hard and soft tissues. Despite these methodological differences, the results were consistent with those of the other studies.

Manual (caliper) measurement of the width of the alveolar bone in the extraction site was used only by Isola et al. (18) who found no significant differences between the study and control groups. They also used free gingival grafts (FGG) for closure of the extraction wound in both groups. Using ADM with FGG resulted in greater new vital bone formation, a higher amount of newly formed bone, and smaller dimensional changes in the bone than FGG alone. This solution appears to be efficient; on the other hand, the need for additional surgery to harvest the FGG brings discomfort to the patient and increases the time demands and, therefore, costs. Hence, the pros and cons of this technique remain debatable.

It is worth noting that, based on our inclusion criteria, all studies compared in this review discussed socket preservation in locations suitable for prosthetic rehabilitation by dental implants. This is mainly because the simple and ethical method of acquiring the bone biopsy during dental implant placement (16–20) is prevented in the case of third molars (as implant placement is rarely indicated after their extraction). This, of course, does not mean that ADM or another augmentation technique should not be considered for third molars—quite on the contrary, there is no reason why this technique should not be equally suitable for promoting the healing processes and reducing bone loss even after third molar extraction (22). Mazzucchi et al. actually reported that autologous dentin grafts can be useful in preventing the development of periodontal defects after surgical extraction of the third molars (23). Similarly, Nguyen reported that grafting the autologous demineralized dentin matrix into the tooth socket after the lower third molar surgery improved wound healing while not increasing the occurrence of postoperative complications compared to teeth extracted without augmentation (24).

Five out of the six selected studies focused on single-rooted teeth without any signs of severe periodontal disease. From both these perspectives, the study by Yang et al. (17) was exceptional as the authors specifically focused on the effect of ADM in molars affected by periodontal disease. They observed significantly better healing in the ADM group than in the control group: only in 1 out of 16 sockets required additional augmentation during implant placement in the ADM group, compared to 5 out of 16 in the control group. These results suggest that the use of ADM for socket preservation in periodontally compromised teeth may be a promising strategy to reduce the need for additional surgical interventions in the future. This result is also corroborated by the outcomes reported by Kim et al. (25) who investigated the use of various grafting materials for ridge preservation in periodontally compromised extraction sites. In their study, the overall success rate of socket healing with augmentation reached 99.3%, indicating that ridge preservation techniques can be highly effective even in compromised periodontal conditions.

Limitations of this narrative review include (besides its narrative character, although supplemented with many features required for systematic review) include the inclusion only of studies in English and a relatively small number of included studies, suffering also from relatively modest numbers of patients and the fact that none of them could have been assessed as a study with a low risk of bias. The heterogeneity among studies from the clinical perspective as well as in endpoint evaluation and short follow-up periods are additional limitations. Lastly, we have included only papers from the last ten years in the analysis, which, theoretically, might lead to missing out on older studies. However, as discussions on autogenous dentin application has only arisen in recent years, searching for older studies would in all likelihood only increase the laboriousness of the identification of suitable studies while not adding any studies of value in this respect.

The strengths of this study lie in maximizing the homogeneity by using only studies with a clearly defined comparator (spontaneous healing), discussion of both histological and radiographic outcomes, and practical clinical framing.

## 5 Conclusion

The use of autogenous dentin as a grafting material for socket preservation appears to be a highly promising approach, at least in the short term, although larger and longer-term studies are needed. The advantages of this approach include very high dimensional stability of the alveolar ridge, support for the healing process, and prevention of the need to use xenografts. On the other hand, the time spent on the preparation of ADM and the need for specialized equipment (grinder for ADM preparation) may pose practical limitations for the wide use in everyday clinical practice. In view of the still relatively low amount and small patient groups, additional studies with larger cohorts (ideally comparing multiple socket preservation techniques) are still needed. Future studies might also benefit from the use of AI in obtaining more accurate measurements from CBCT imagery, which might help in objectivizing and improving accuracy of these measurements.
